# Relationship of Melatonin Levels in Blood and Urine with Sleep Quality in Children Admitted to Pediatric Intensive Care Unit

**DOI:** 10.3390/children12081074

**Published:** 2025-08-15

**Authors:** Miriam García-San Prudencio, Gema Manrique, Raquel Cieza, Cristina Corraliza, Patricia Arias, Elena Medina, Jesús López-Herce, Santiago Mencía

**Affiliations:** 1 Pediatric Intensive Care Department, Gregorio Marañón General University Hospital, 28009 Madrid, Spainraquel.cieza@salud.madrid.org (R.C.); patricia.arias@quironsalud.es (P.A.);; 2 Public Health and Mother-Child Department, School of Medicine, Complutense University of Madrid, 28040 Madrid, Spain; 3 Gregorio Marañón Health Research Institute, Mother-Child Health and Development Network (Red SAMID) of ISCIII-Sub-Directorate General for Research Assessment and Promotio and the European Regional Development Fund (ERDF), ref RD16/0026/0007, 28029 Madrid, Spain

**Keywords:** PICU, melatonin, sleep, 6-sulphatoxymelatonin

## Abstract

**Highlights:**

**What are the main findings?**

- Children admitted to the PICU frequently fail to achieve the recommended amount of sleep for their age.
- Altered serum and urinary melatonin levels are significantly associated with poorer sleep quality.

**What is the implication of the main findings?**

- In the PICU, circadian rhythm is disrupted. Among modifiable factors that contribute to this, are the environmental factors such as artificial lighting, noise, and nocturnal interventions.
- Melatonin is a potential non-invasive biomarker of circadian dysfunction in the PICU setting.

**Abstract:**

Objectives: Melatonin regulates the sleep–wake cycle, which may be altered in children admitted to the pediatric intensive care unit (PICU). The aim is to analyze whether blood and urine melatonin levels are related to sleep quality in children admitted to the PICU. Methods: A single-center, prospective observational study was conducted at the PICU in a tertiary referral center in Madrid, Spain. It included patients (1 month–16 years) admitted to the PICU. Results: A total of 40 children were included in the study (52.5% male), analyzing 2 days for each patient (80 total study days). The median age of the patients was 34.5 months. The median number of hours of sleep over the whole day was 9.9 h (IQR 8.4–12.6); nighttime was 7.5 h (IQR 6.5–9) and daytime was 2.5 h (IQR 1.4–5.7). Only 8.5% of patients slept the appropriate number of hours of nighttime sleep for their age. High serum melatonin levels in the morning were correlated with more nocturnal awakenings (r = 0.35, *p* = 0.01) and less continuous sleep time (r = −0.29, *p* = 0.03). Altered urine levels in the morning correlated with shorter 24 h total sleep time (r = −0.43, *p* = 0.01). Inadequate urine levels at 7 pm correlated with a shorter duration of continuous nocturnal sleep (r = −0.37, *p* = 0.03). Conclusions: Children admitted to the PICU often do not achieve the recommended hours of sleep for their age. A significant percentage of these children exhibit an altered melatonin secretion pattern, which is associated with shorter nighttime sleep duration and longer daytime sleep duration.

## 1. Introduction

Sleep is a fundamental neurophysiological process essential for sustaining life and ensuring appropriate physical and psychological development in children [1,2]. In the pediatric intensive care unit (PICU), sleep quality is frequently compromised and is known to be influenced by both environmental conditions and routine clinical care activities [3,4,5,6,7].

Sleep follows a structured organization in the form of recurrent cycles, collectively constituting the sleep–wake cycle. This cycle is regulated by a combination of endogenous mechanisms and exogenous stimuli [1,2,8], with exposure to natural light and darkness playing a pivotal role. These light–dark cues modulate the secretion of melatonin, a hormone that governs both the initiation of sleep and the synchronization of circadian rhythms. Melatonin production follows a diurnal pattern and is evolutionarily conserved across mammalian species [9].

Melatonin is predominantly metabolized in the liver (85–90%) through hydroxylation and subsequent conjugation with sulfate or glucuronic acid. Its principal urinary metabolite, 6-sulfatoxymelatonin (6-SMEL), reflects circulating melatonin levels and serves as a reliable biomarker for melatonin secretion [10,11]. Studies in critically ill adults have demonstrated the absence of normal circadian variation in urinary 6-SMEL excretion, with levels remaining relatively stable throughout the 24 h period [12].

The objective of this study was to determine whether blood and urinary melatonin levels are associated with sleep quality in children admitted to the PICU.

## 2. Materials and Methods

A prospective observational study was conducted on critically ill pediatric patients aged between 1 month and 16 years, who were admitted to the pediatric intensive care unit (PICU) for more than 48 h. Patients receiving intravenous infusions of sedatives, analgesics, or neuromuscular blocking agents, those treated with melatonin, and those with pre-existing neurological disorders were excluded from the study. The research protocol was approved by the institution’s local ethics committee.

Data collection was performed using a structured form completed by the nursing staff or attending physicians during each shift (morning (8 am to 3 pm), afternoon (3 pm to 10 pm), and night (10 pm to 8 am)) over a 48 h observation period. The form captured the following variables:

- Patient characteristics: demographic data, location within the PICU (room type), clinical interventions received, and medications administered during the study period, including enteral abstinence medication.
- Sleep characteristics: total sleep duration during the day and night (including naps), the duration of continuous nighttime sleep episodes, and characteristics of sleep interruptions (frequency, duration, and causes). The sleep data were obtained through clinical observation and routine nursing documentation recorded in patient charts. Sleep quality was categorized as either *adequate* or *inadequate* based on whether the patient met the 2nd and 50th percentiles of expected sleep duration for their age, according to the normative values published by Iglowstein et al. [13].
- Environmental factors were assessed through objective measurements of noise and light levels:
  ○ Noise was measured continuously using the *PCE-322A^®^* sound level meter, which operates within a range of 30 to 130 decibels (dB) and offers a measurement accuracy of ±1.4 dB.
  ○ Light intensity was measured every two hours using the *PCE-174^®^ lux meter*, with a measuring range of 400 to 4000 lux and an accuracy of ±5% for levels below 10,000 lux and ±10% for levels above 10,000 lux.
- Melatonin levels were evaluated through the collection of biological samples on the first day of observation. Specifically:
  ○ Blood and urine samples were collected early in the morning (around 7:00 a.m. or upon the patient’s awakening).
  ○ An additional urine sample was obtained in the late afternoon (between 7:00 and 8:00 p.m.).

Blood samples were centrifuged at 3000 revolutions per minute (rpm) for 10 min to separate the serum. Urine samples from non-continent patients were collected using adhesive urine collection bags. Both serum and urine samples were stored at −80 °C until transportation to an external reference laboratory, ensuring the maintenance of the cold chain throughout the process.

Alterations in melatonin secretion were classified according to the following criteria:

- Elevated morning serum melatonin: Values > 30 pg/mL at 7:00 a.m. were considered abnormal. At this time of day, melatonin levels are physiologically low. Elevated levels suggest a disruption in the normal circadian secretion pattern of melatonin.
- Reduced urinary 6-SMEL at 7:00 a.m.: Concentrations < 58 mcg/L were considered low. Morning urine typically contains melatonin metabolites accumulated during nighttime sleep; therefore, reduced levels are indicative of decreased nocturnal melatonin secretion.
- Elevated urinary 6-SMEL at 7:00 p.m.: Concentrations > 58 mcg/L were considered elevated. Since this sample reflects melatonin metabolism during the daytime, lower levels would be expected. Elevated values may suggest abnormal daytime melatonin secretion, which could be associated with increased daytime sleepiness or circadian rhythm dysregulation.

### Statistical Analysis

Statistical analysis was performed with SPSS version 25 (SPSS Inc., Chicago, IL, USA). The normal distribution of quantitative variables was assessed with the Kolmogorov Smirnov test and they were expressed as mean and standard deviation or median and interquartile range according to their distribution. To compare qualitative variables, the Chi-square test was used, or Fisher’s test when the expected percentage of frequencies less than 5 exceeded 20%, with a confidence interval of 95%. For the comparison of quantitative variables, Student’s or Mann–Whitney U tests were used depending on whether their distribution was normal or not. Correlation between continuous variables was calculated using Pearson’s correlation coefficient, being considered a very high correlation when >0.7, high > 0.5, moderate from 0.3 to 0.5, and low < 0.3. Multivariate analysis (linear regression for quantitative dependent variables or logistic regression for qualitative dependent variables) was performed to study the association of personal factors, medical conditions, medications, light, noise, and sleep quality on melatonin levels. A value of *p* ≤ 0.05 was considered statistically significant.

## 3. Results

### 3.1. Patient Characteristics

A total of 40 critically ill children were included in the study, with data collected over two consecutive days for each patient, resulting in 80 total study days of observation. The median age was 34.5 months (interquartile range [IQR]: 4.3–118.3), and 47.5% of the participants were female. The majority of patients (80%) had underlying congenital or acquired heart disease. At the time of the sleep assessment, the median duration of the PICU stay was 7.5 days (IQR: 4–14.3).

Sedation and neurological status assessments yielded the following median scores: Comfort-B scale, 18.5 (IQR: 15–21); analgesia scale, 0 (IQR: 0–2); Sophia withdrawal scale, 1 (IQR: 0–2); and Cornell Assessment of Pediatric Delirium (CAPD) scale, 0 (IQR: 0–1.5).

Regarding room allocation, 46.3% of patients were admitted to a large shared room with six beds, 36.3% to a smaller shared room with three beds, and 17.5% to individual rooms.

In terms of pharmacological management, 47.3% of patients received oral sedative or analgesic medications during the study period. The most commonly used medications were clorazepate (35.5%), methadone (32.9%), clonidine (26.3%), chlorpromazine (13.1%), and levomepromazine (5.3%). These medications were administered either as a continuation of previously suspended intravenous treatments or prophylactically to prevent withdrawal syndrome or delirium. Of these, 10.5% received a single drug, while 36.8% were administered two or more sedative/analgesic agents. In the multivariate logistic regression analysis, treatment with enteral sedoanalgesic medication was not a statistically significant factor related to sleep duration (Table A1).

### 3.2. Sleep Characteristics

The median total sleep duration over a 24 h period was 9.9 h (IQR: 8.4–12.6). Of this, the median nighttime sleep duration was 7.5 h (IQR: 6.5–9), while daytime sleep accounted for a median of 2.5 h (IQR: 1.4–5.7). The median duration of continuous nighttime sleep was 240 min (IQR: 152–300).

Only 8.5% of the patients achieved the age-appropriate amount of nighttime sleep, whereas 37% met the recommended total daily sleep duration for their age, predominantly due to increased daytime sleep. Nighttime family presence was documented in 48.6% of cases.

### 3.3. Melatonin Levels in Blood and Urine

Table 1 presents the melatonin measurements obtained from the study population, along with reference values expected in healthy individuals.

When analyzed by age groups, patients under 1 year of age had median blood melatonin levels of 110 pg/mL (IQR 70.5–110), a morning 6-SMEL of 18.9 mcg/L (IQR 11.75–22.3), and an evening 6-SMEL of 13.9 mcg/L (IQR 8.65–17.28). Those between 1 and 9 years old had median blood melatonin levels of 98 pg/mL (IQR 70.75–110), a morning 6-SMEL of 36.9 mcg/L (IQR 16.62–68.27), and an evening 6-SMEL of 76.7 mcg/L (IQR 16.2–90.15). Those over 10 years old had median blood melatonin levels of 98 pg/mL (IQR 27–110), a morning 6-SMEL of 20.4 mcg/L (IQR 13.9–50.8), and an evening 6-SMEL of 15.2 mcg/L (IQR 8.20–76.7). There were no significant differences in serum melatonin levels among the three age groups (*p* = 0.36) and evening 6-SMEL (*p* = 0.94). However, morning 6-SMEL levels did show significant differences between the three groups (*p* = 0.26).

At 7:00 a.m., 69% of patients exhibited elevated serum melatonin concentrations, while 60% showed reduced urinary 6-SMEL levels. Additionally, 21% of patients had elevated 6-SMEL levels in urine collected at 7:00 p.m.

Correlation analyses revealed a weak but significant association between age and both serum melatonin (r = 0.27, *p* = 0.04) and morning urinary 6-SMEL (r = 0.43, *p* < 0.01). No significant correlation was observed between the PRISM III severity score and melatonin levels. However, a longer duration of prior mechanical ventilation (MV) was associated with higher 6-SMEL levels in the evening sample (r = 0.45, *p* = 0.01), while a longer PICU length of stay prior to data collection was correlated with lower serum melatonin (r = −0.27, *p* = 0.04) and lower morning 6-SMEL levels in urine (r = −0.55, *p* = 0.01).

Multivariate analysis showed that underlying heart disease and a history of surgery were associated with altered serum melatonin concentrations. Age was significantly associated with alterations in both morning and evening urinary 6-SMEL levels. Additionally, altered morning 6-SMEL levels were associated with a longer PICU stay and prior mechanical ventilation (Table 2).

**Figure 1:**
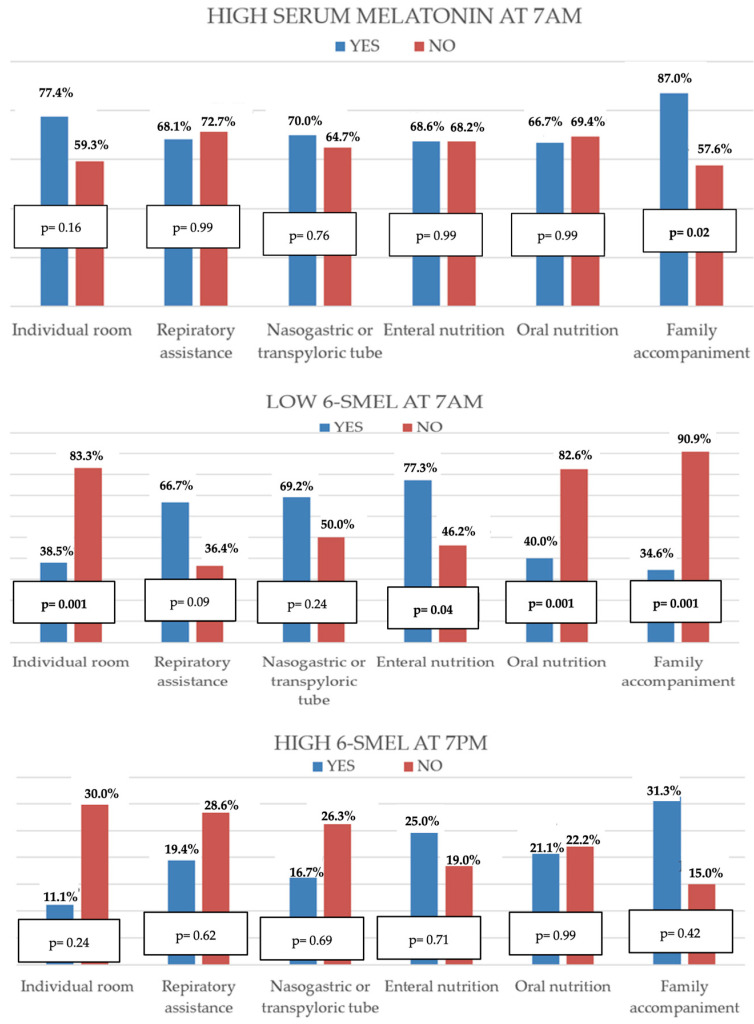
Comparison of the percentage of patients with altered melatonin levels in relation to clinical care characteristics. 6-SMEL = 6-sulphatoxymelatonin.

As illustrated in Figure 1, patients accompanied by family members more frequently exhibited elevated serum melatonin. Lower morning urinary 6-SMEL levels were more prevalent in children in shared rooms, those receiving enteral nutrition, and those not accompanied by a caregiver during sleep. No significant associations were identified between clinical care variables and evening 6-SMEL levels.

Multivariate analysis confirmed the associations with serum melatonin and evening 6-SMEL alterations. Furthermore, altered morning 6-SMEL levels were independently associated with both the type of room and presence of family accompaniment during the night (Table 3).

### 3.4. Relationship Between Melatonin Levels and Sleep Characteristics

Elevated serum melatonin concentrations at 7:00 a.m. were associated with a greater number of nocturnal awakenings and a reduction in continuous nighttime sleep duration. Similarly, higher 6-SMEL levels at 7:00 a.m. were correlated with shorter daytime sleep duration and reduced total 24 h sleep time.

In contrast, elevated 6-SMEL levels at 7:00 p.m. were linked to increased nocturnal awakenings, shorter uninterrupted nighttime sleep, and reduced overall daily sleep time (Table A2).

The presence of melatonin secretion disturbances and their relationship to expected sleep duration (based on the 2nd and 50th percentiles of age-adjusted sleep duration as per Iglowstein et al. [13]) revealed distinct patterns (Table A3):

- Altered serum melatonin was associated with shorter nighttime sleep duration, more frequent awakenings, shorter total nighttime sleep, and reduced continuous sleep episodes.
- Low morning 6-SMEL levels were associated with longer daytime sleep and increased total 24 h sleep.
- High evening 6-SMEL was associated with reduced total daily sleep duration and a higher number of nighttime awakenings.

**Figure A1:**
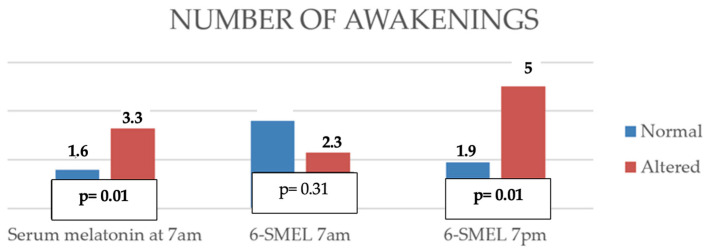
Relationship of the number of awakenings to altered serum and urine melatonin levels. 6-SMEL = 6-sulphatoxymelatonin.

**Figure A2:**
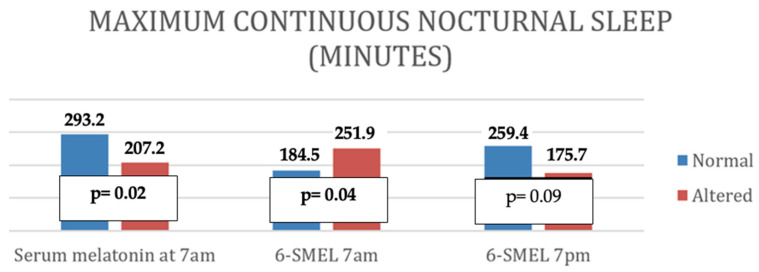
Relationship of maximum continuous nocturnal sleep time to serum and urine melatonin levels. 6-SMEL = 6-sulphatoxymelatonin.

**Figure A3:**
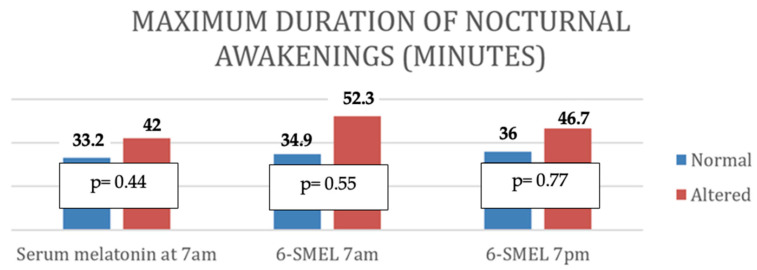
Relationship of maximum duration of nocturnal awakenings to serum and urine melatonin levels. 6-SMEL = 6-sulphatoxymelatonin.

**Figure A4:**
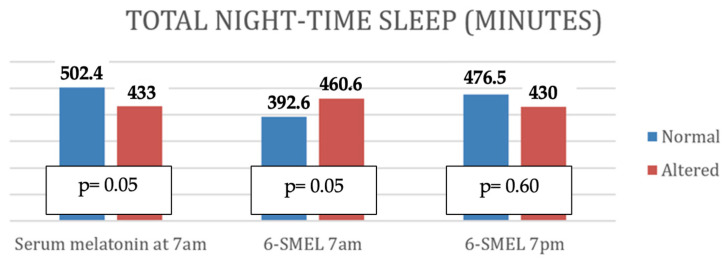

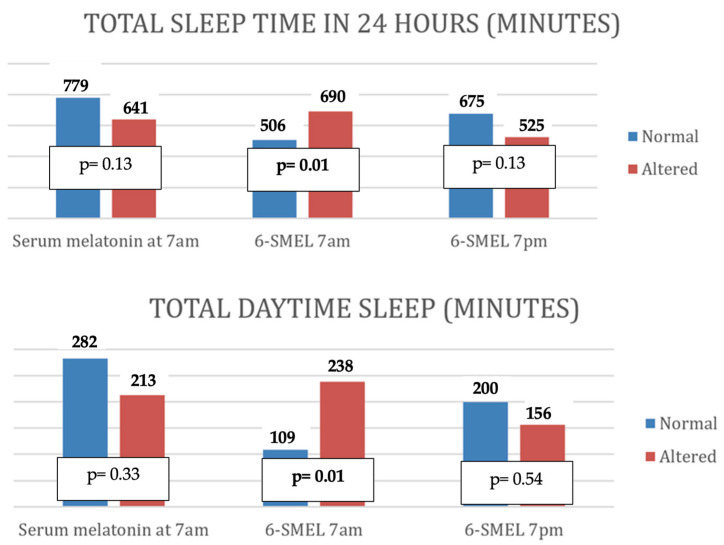
Relationship of total night-time sleep, total sleep time in 24 h and total daytime sleep to serum and urine melatonin levels. 6-SMEL = 6-sulphatoxymelatonin.

These associations are further illustrated in Figure A1, Figure A2, Figure A3 and Figure A4.

In the multivariate analysis (Table 4), no statistically significant relationships were observed between serum melatonin or evening urinary 6-SMEL levels and total sleep duration. However, low morning 6-SMEL levels were significantly associated with shorter nighttime sleep duration (2nd percentile threshold, *p* = 0.03).

### 3.5. Relationship Between Melatonin Levels and Environmental Factors

**Figure 2:**
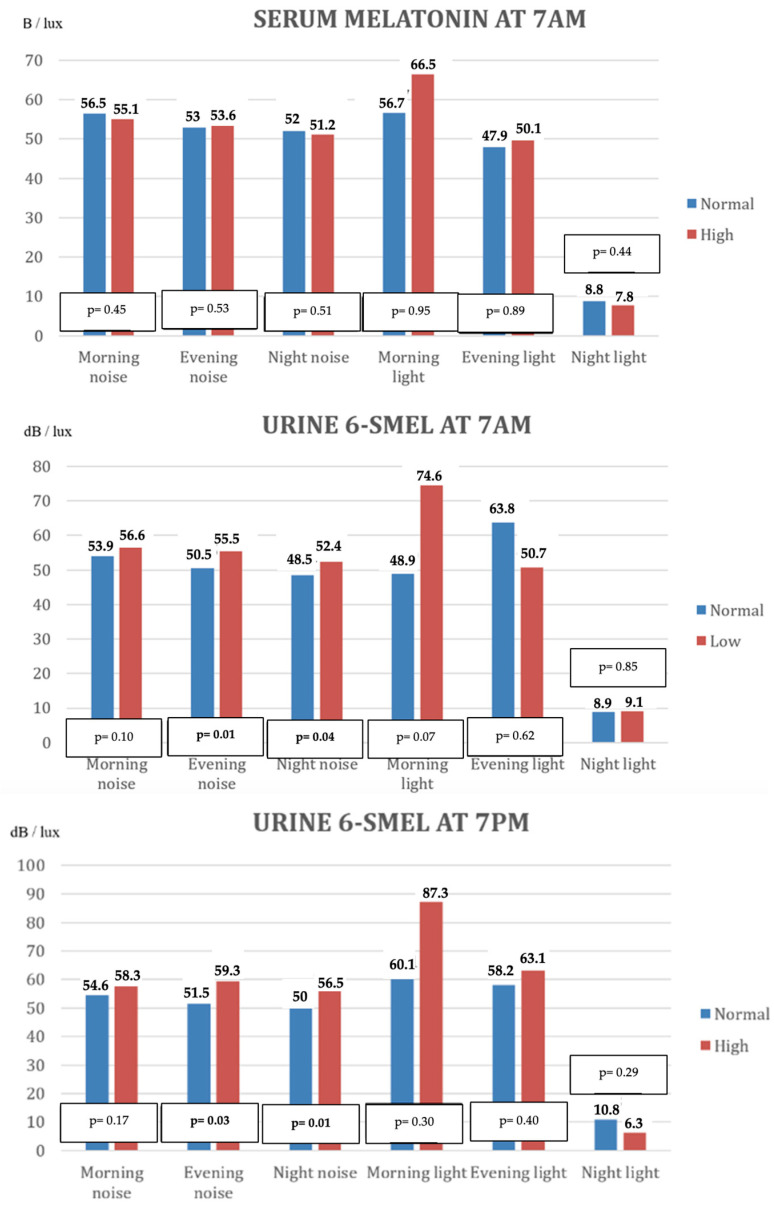
Mean values of noise (dB) and light (lux) in each shift in relation to the alteration of serum and urine melatonin values in the morning and evening. 6-SMEL = 6-sulphatoxymelatonin.

The association between melatonin levels and environmental parameters—ambient noise and light levels—recorded during each nursing shift is depicted in Figure 2 and Table A4.

No statistically significant relationship was observed between serum melatonin concentrations at 7:00 a.m. and the levels of noise or light recorded. However, elevated noise levels during evening and night shifts were significantly associated with altered urinary 6-SMEL concentrations, both in the morning (7:00 a.m.) and evening (7:00 p.m.) samples.

In the multivariate analysis, a significant association was identified between morning shift light intensity and disturbances in 6-SMEL levels at 7:00 a.m. (Table 5). Additionally, a significant relation was found between the 24 h average noise level and both serum melatonin concentrations and morning urinary 6-SMEL levels (Table 6).

## 4. Discussion

Although previous studies have investigated sleep patterns in hospitalized pediatric populations [3,4,5,6,7], to our knowledge, this is the first study to evaluate serum and urinary melatonin levels in critically ill children admitted to a PICU and to explore their relationship with sleep quality, as well as clinical and environmental factors.

Our findings indicate that nocturnal melatonin secretion is frequently impaired in this population. Furthermore, alterations in melatonin levels—both in blood and in urinary metabolites—were significantly associated with disturbed sleep architecture.

### 4.1. Melatonin Levels in Blood and Urine

Melatonin is a hormone with somnogenic properties, secreted in alignment with the circadian sleep–wake cycle [14]. Under normal physiological conditions, melatonin secretion begins to increase during the evening (around 7–8 pm) and is inhibited by light exposure at dawn. Reference values for melatonin and its urinary metabolite 6-SMEL have been established by several authors [15,16]; however, considerable variability has been reported, influenced by factors such as age, geographic location (daylight exposure), lifestyle patterns, and comorbidities. This interindividual variability is particularly pronounced in pediatric populations [17], and is further amplified in critically ill children, whose circadian rhythms are often profoundly disrupted in the intensive care setting [12,18,19,20].

Respecting serum melatonin, normal day levels in healthy children are typically <10 pg/mL [21,22], but there are studies [23] that report mean morning serum melatonin levels as high as 40 pg/mL. However, there is no clear threshold in the literature regarding normal morning levels in pediatric critically ill patients. For our study, we followed the reference values proposed by the laboratory, due to the lack of literature establishing a clear threshold in critically ill pediatric patients, given the variability in values reported in previous studies.

Most studies have favored the measurement of 6-SMEL in urine as a surrogate for circulating melatonin, due to the non-invasive nature of this method and its feasibility in pediatric populations without intravenous access [24,25].

Age is one of the factors that may contribute to variability in melatonin secretion in a healthy population, especially with regard to nocturnal melatonin secretion. In our study, there are significant differences in morning 6-SMEL among age groups. However, recent studies have reported that the daily excretion of 6-SMEL and morning serum melatonin levels remains stable until adolescence [20,23]; therefore, it requires further investigation.

In the present study, we performed a single measurement of serum melatonin at 7 a.m.—when circulating melatonin levels should physiologically be low—and two measurements of 6-SMEL: one at 7 a.m. (reflecting overnight secretion) and another at 7 p.m. (when metabolite levels should be minimal due to suppressed daytime secretion).

When melatonin values were categorized as altered based on expected physiological thresholds, the most frequently disturbed parameter was elevated serum melatonin at 7 a.m., followed by abnormally low urinary 6-SMEL at the same time point. These results suggest that nocturnal melatonin secretion is markedly impaired in children admitted to the PICU. Notably, although a significant proportion of patients exhibited increased daytime sleep, elevated 6-SMEL concentrations at 7 p.m. were observed in only 21% of the cohort.

These findings are consistent with previous reports by Foster [20] and Mundigler [12], which support the notion that critically ill children experience a loss of circadian melatonin rhythmicity. This is evidenced by elevated morning serum melatonin levels and diminished 6-SMEL excretion both in the morning and late afternoon. Such disruption may indicate a shift from a nocturnally peaked secretion pattern to a flattened, continuous secretion throughout the day, potentially contributing to excessive daytime sleepiness and reduced nocturnal sleep consolidation.

### 4.2. Relationship Between Melatonin Levels and Clinical Patient Characteristics

Children admitted to shared rooms and those accompanied by family members during sleep exhibited more pronounced alterations in melatonin secretion, findings that correlate with poorer nocturnal sleep quality. These results are consistent with previous studies [3,4,7,26,27,28], emphasizing the importance of admitting critically ill pediatric patients in individual rooms that promote restful sleep and minimize environmental disturbances.

Furthermore, children receiving enteral nutrition exhibited a higher frequency of abnormalities in morning urinary 6-SMEL levels. This observation may be explained by the concept of chrononutrition [29,30,31], wherein artificial feeding regimens disrupt the physiological timing of nocturnal melatonin secretion. The mechanical interventions associated with feeding tubes and pumps, as well as deviations from normal feeding schedules, may alter circadian regulation, resulting in decreased nocturnal sleep and increased daytime sleepiness. However, due to an insufficient sample size, the study was unable to differentiate between intermittent and continuous enteral feeding modalities.

Additionally, patients receiving enteral sedoanalgesic medications also exhibited greater disturbances in both morning and evening 6-SMEL levels, although the use of these medications was not a statistically significant factor related to sleep duration.

### 4.3. Relationship Between Melatonin Levels and Sleep Characteristics

An analysis of the association between altered melatonin levels and sleep characteristics revealed that decreased nocturnal melatonin secretion, as indicated by low 6-SMEL levels in morning urine, was correlated with a reduced duration of nighttime sleep. Conversely, children exhibiting abnormally elevated serum melatonin levels in the morning also demonstrated shorter nighttime sleep duration.

These findings support the hypothesis that the disruption or absence of the normal nocturnal melatonin peak is associated with impaired nocturnal sleep quality. Furthermore, higher morning 6-SMEL levels, reflecting adequate nocturnal melatonin secretion, appear to correlate with reduced daytime sleepiness. Similarly, elevated 6-SMEL levels measured at 7 p.m.—indicative of increased daytime melatonin secretion—were associated with shorter nighttime sleep, alongside increased daytime sleepiness.

### 4.4. Relationship Between Melatonin Levels and Ambient Light and Noise

In this study, increased noise levels during the afternoon and evening shifts were associated with elevated urinary 6-SMEL concentrations at 7 pm, concomitant with increased daytime sleepiness. This finding likely reflects the impact of nocturnal noise disturbances on sleep quality, resulting in compensatory daytime somnolence. These results align with previous research demonstrating the detrimental effects of excessive noise on sleep patterns [32].

Regarding ambient light exposure, a negative correlation was observed between morning light intensity and 6-SMEL levels measured at 7 am. Contrary to expectations—where adequate daytime light exposure should promote higher nocturnal melatonin secretion—our findings suggest that other factors, such as insufficient artificial light exposure during daytime hours, may contribute to the observed reduction in nocturnal melatonin secretion.

### 4.5. Limitations

This study was conducted at a single center, resulting in all patients being exposed to the same environmental conditions. These conditions may differ in other PICUs, highlighting the need for similar studies in diverse settings to determine whether the findings are generalizable.

Critically ill patients are subject to multiple concurrent environmental and clinical factors, interventions, and medications that influence sleep duration and characteristics. Although multivariate regression analyses were performed, it is not possible to definitively establish causal relationships between individual factors and sleep disturbances.

Melatonin levels were measured once a day and 6-SMEL only at two specific times during the day. A comprehensive assessment of the melatonin secretion profile would require sampling at multiple time points across the 24 h cycle; however, this was not feasible due to the increased invasiveness, labor intensity, and cost. Furthermore, the absence of objective sleep measures is another limitation of this study.

Another limitation of this study is the use of uniform threshold values for interpreting serum melatonin and urinary 6-SMEL levels across a broad pediatric age range. Melatonin production is known to vary significantly with age, particularly in early infancy and during adolescence. Nevertheless, it is important to note that the majority of the study population was between 2 and 9 years old, an age range in which melatonin production is typically more stable. While the use of fixed thresholds may not fully reflect developmental differences, we believe this limitation has minimal impact on the main findings. Future studies should consider age-specific reference values and stratified analyses to better account for maturational changes in melatonin physiology.

Finally, the study did not evaluate the evolution of sleep quality following PICU discharge, limiting the understanding of whether sleep disturbances persist or improve after hospitalization.

## 5. Conclusions

Children admitted to the PICU experience a reduction in the duration of sleep during the nighttime hours and fail to reach the recommended hours of sleep for their age. Conversely, they exhibit increased daytime sleep duration.

A significant proportion of these children show altered melatonin secretion patterns, characterized by abnormally high serum melatonin and abnormally low 6-SMEL levels at 7 a.m., as well as low 6-SMEL levels in the late afternoon. These alterations are associated with shorter nighttime sleep duration.

## Figures and Tables

**Table 1:** Melatonin (serum) and 6-SMEL ^1^ (urine) levels.

	Number of Determinations	Melatonin Levels (Median)	Normal Reference Values
**Serum 7 am (pg/mL)**	58	110 (IQR 25.8–110)	<30
**Urine 6-SMEL ^1^** **7 am (mcg/L)**	50	40.3 (IQR 16.6–125.6)	>58
**Urine 6-SMEL ^1^** **7 pm (mcg/L)**	38	15.2 (IQR 8.2–25.7)	<58

^1^ 6-SMEL: 6-sulphatoxymelatonin.

**Table 2:** Multivariate study of the relationship between altered melatonin levels and patient characteristics.

	Serum 7 AM ↑	6-SMEL ^1^ 7 AM ↓	6-SMEL ^1^ 7 PM ↑
**AGE (months)**	*p* = 0.08	***p* = 0.05 (ß—0.33)**	***p* = 0.02 (ß 0.52)**
**Sex**	*p* = 0.16	*p* = 0.07	*p* = 0.74
**Time in PICU at the beginning of study**	*p* = 0.51	***p* = 0.02 (ß 0.94)**	*p* = 0.86
**Heart disease**	***p* = 0.02 (ß—0.45)**	*p* = 0.08	*p* = 0.75
**Previous surgery**	***p* = 0.04 (ß 0.37)**	*p* = 0.15	*p* = 0.10
**PRISM III**	*p* = 0.82	*p* = 0.86	*p* = 0.18
**Prior MV ^2^**	*p* = 0.07	***p* = 0.03 (ß 0.48)**	*p* = 0.56
**Days with previous MV**	*p* = 0.65	*p* = 0.12	*p* = 0.86

^1^ 6-SMEL = 6-sulphatoxymelatonin. ^2^ MV = mechanical ventilation.

**Table 3:** Multivariate study of the relationship between patients’ medical conditions and altered melatonin levels.

	Serum 7 AM ↑	6-SMEL ^1^ 7 AM ↓	6-SMEL ^1^ 7 PM ↑
**Shared room**	*p* = 0.11	***p* = 0.001**	*p* = 0.20
**Respiratory assistance**	*p* = 0.24	*p* = 0.31	*p* = 0.39
**Enteral nutrition**	*p* = 0.50	*p* = 0.29	*p* = 0.24
**Family accompaniment**	***p* = 0.03**	***p* = 0.001**	*p* = 0.40

^1^ 6-SMEL = 6-sulphatoxymelatonin.

**Table 4:** Multivariate analysis of the relationship between altered serum and urine melatonin levels and sleep duration.

	Serum 7 AM ↑	6-SMEL ^1^ 7 AM ↓	6-SMEL ^1^ 7 PM ↑
Nighttime sleep p2	*p* = 0.17	***p* = 0.03**	*p* = 0.69
Nighttime sleep p50	*p* = 0.99	*p* = 0.99	*p* = 0.99
Daytime sleep p2 *	-	-	-
Daytime sleep p50	*p* = 0.99	*p* = 0.99	*p* = 0.99
Sleep in 24 h p2	*p* = 0.99	*p* = 0.99	*p* = 0.99
Sleep in 24 h p50	*p* = 0.99	*p* = 0.99	*p* = 0.85

^1^ 6-SMEL = 6-sulphatoxymelatonin. * No patients were classified as poor daytime sleep quality p2.

**Table 5:** Multivariate analysis of the relationship between noise and light levels in each shift and alterations in melatonin levels in blood and urine.

	High Serum Melatonin At 7 AM	Low 6-Smel ^1^ At 7 AM	High 6-Smel ^1^ At 7 PM
Morning Noise	*p* = 0.53	*p* = 0.89	*p* = 0.54
Evening Noise	*p* = 0.65	*p* = 0.21	*p* = 0.17
Night Noise	*p* = 0.96	*p* = 0.30	*p* = 0.15
Morning Light	*p* = 0.25	***p* = 0.05**	*p* = 0.64
Evening Light	*p* = 0.75	*p* = 0.08	*p* = 0.44
Night Light	*p* = 0.76	*p* = 0.42	*p* = 0.12

^1^ 6-SMEL = 6-sulphatoxymelatonin.

**Table 6:** Multivariate analysis of the relationship between daily noise and light levels and alterations in melatonin levels in blood and urine.

	Medium Noise in 24 h	Medium Light in 24 h
High Serum Melatonin at 7 AM	***p* = 0.04**	*p* = 0.50
Low 6-SMEL ^1^ at 7 AM	***p* = 0.02**	*p* = 0.99
High 6-SMEL ^1^ at 7 PM	*p* = 0.96	*p* = 0.99

^1^ 6-SMEL = 6-sulphatoxymelatonin.

## Data Availability

Data are unavailable due to privacy or ethical restrictions.

## Abbreviations

The following abbreviations are used in this manuscript:

PICU	Pediatric Intensive Care unit
6-SMEL	6-sulfatoxymelatonin
CAPD	Cornell Assessment of Pediatric Delirium
PRISM	Pediatric Risk of Mortality
IQR	Interquartile Range
MV	Mechanical Ventilation
SD	Standard Deviation

## Appendix A

**Table A1:** Multivariate analysis of the relationship between sleep duration and enteral sedoanalgesic treatment.

	Good Sleep			
	Nighttime p2	Nighttime p50	24 h p2	24 h p50
**Enteral sedoanalgesic medication**	*p* = 0.90	*p* = 0.16	*p* = 0.52	*p* = 0.93
**Drug combination**	*p*=0.48	*p* = 0.22	*p* = 0.08	*p* = 0.49

**Table A2:** Correlation between serum and urine melatonin levels and sleep characteristics.

		Serum Melatonin 7 am	6-SMEL ^1^ 7 am	6-SMEL ^1^ 7 pm
**Number of awakenings**	*p*	**0.01**	0.10	**0.01**
	r	**0.35**		**0.46**
**Total time of nighttime sleep (minutes)**	*p*	0.08	0.07	0.08
	r			
**Maximum time of continuous sleep (minutes)**	*p*	**0.03**	0.10	**0.03**
	r	**−0.29**		**−0.37**
**Maximum duration of awakenings (minutes)**	*p*	0.38	0.73	0.97
	r			
**Sleep in 24 h (minutes)**	*p*	0.14	**0.01**	**0.04**
	r		**−0.43**	**−0.38**
**Daytime sleep (minutes)**	*p*	0.37	**0.03**	0.37
	r		**−0.34**	

^1^ 6-SMEL = 6-sulphatoxymelatonin. r = correlation coefficient.

**Table A3:** Comparison of the number of patients with good or poor sleep quality when their blood or urine melatonin levels were altered.

Sleep Quality		Altered Melatonin					
		Serum 7 a.m.	*p*	Urine 7 a.m.	*p*	Urine 7 p.m.	*p*
**Nighttime p2**	B	20	0.09	12	0.08	3	1
	G	16		14		3	
**Nighttime p50**	B	35	**0.01**	21	0.06	5	1
	G	1		5		1	
**Daytime p2**	B *	-		-		-	
	G	34		26		8	
**Daytime p50**	B	2	0.57	2	0.52	1	0.46
	G	32		24		7	
**24 h p2**	B	8	1	5	0.08	4	**0.02**
	G	23		18		2	
**24 h** **p50**	B	21	0.06	11	**0.02**	4	1
	G	10		12		2	

B: bad; G: good. * No patients were classified as poor daytime sleep quality p2.

**Table A4:** Mean values and standard deviation of noise (dB) and light (lux) for each shift in relation to the alteration of serum and urine melatonin values in the morning and evening.

	Serum Melatonin at 7 AM					
	High			Normal		
	Morning	Evening	Night	Morning	Evening	Night
**Noise** **dB** **(mean values** ± **SD)**	55.1 ± 6.1	53.6 ± 7.2	51.2 ± 6.1	56.5 ± 4.3	53 ± 4.6	52 ± 5.4
**Light lux** **(mean values** ± **SD)**	66.5 ± 49.1	50.1 ± 28.1	7.8 ± 6.9	56.7 ± 13.4	47.9 ± 28.9	8.8 ± 7.7
	**Urine 6-SMEL AT 7 AM**					
	**Low**			**Normal**		
	**Morning**	**Evening**	**Night**	**Morning**	**Evening**	**Night**
**Noise** **dB** **(mean values** ± **SD)**	56.6 ± 6.6	55.5 ± 4.1	52.4 ± 4.6	53.9 ± 4.7	50.5 ± 8.1	48.9 ± 6.6
**Light lux** **(mean values** ± **SD)**	74.6 ± 49.7	50.7 ± 30.2	9.1 ± 7.8	48.9 ± 28.2	63.8 ± 28.6	8.9 ± 6.8
	**Urine 6-SMEL at 7 PM**					
	**High**			**Normal**		
	**Morning**	**Evening**	**Night**	**Morning**	**Evening**	**Night**
**Noise** **dB** **(mean values** ± **SD)**	58.3 ± 4.5	59.3 ± 1.4	56.5 ± 3.1	54.6 ± 6.7	51.5 ± 6.3	50.0 ± 5.7
**Light lux** **(mean values** ± **SD)**	87.3 ± 83.8	63.1 ± 40.1	6.3 ± 3.0	60.1 ± 31.9	58.2 ± 29.1	10.8 ± 8.6

SD = Standard deviation.
